# Is continuous in-line blood gas monitoring reliable during cardiopulmonary bypass when PaO_2_ and PaCO_2_ are calculated rather than measured?

**DOI:** 10.1051/ject/2025051

**Published:** 2026-03-13

**Authors:** Min-Ho Lee, Tami Rosenthal

**Affiliations:** 1 Perfusion Services, Children’s Hospital of Philadelphia 3401 Civic Center Blvd. Philadelphia PA 19104 USA

**Keywords:** Continuous in-line blood gas monitoring, PaO_2_ and PaCO_2_, Measured vs. calculated, Laboratory blood analyzer, Cardiopulmonary bypass

## Abstract

*Background*: The accuracy and precision of continuous in-line blood gas monitoring (CILBGM) are crucial for optimal blood gas management during cardiopulmonary bypass (CPB) and improved patient outcomes. CILBGM devices, such as the CDI 500/550 system, measure PaO_2_ and PaCO_2,_ and B-Capta measures PaO_2_ through direct contact with arterial blood. However, the Quantum perfusion system with Quantum Ventilation_2_ (Quantum System) does not measure but calculates PaO_2_ and PaCO_2_ using several non-invasive sensors and proprietary formulas. We have observed that the calculated in-line PaO_2_ and PaCO_2_ values from Quantum System are frequently significantly higher than those obtained from iSTAT, a point-of-care blood analyzer, exceeding acceptable targets. *Methods*: We conducted a retrospective study involving 81 patients who underwent cardiac surgery using the Quantum System with its own CILBGM and the FX05 oxygenator. The aim was to identify the degree, timing, and possible patterns of error of the calculated in-line PaO_2_ and PaCO_2_. *Results*: Our study showed that the errors of calculated in-line PaO_2_ exceed the acceptable target at the 1st blood gas series and during the rewarming and rewarmed periods, correlating with patient weight. The calculated in-line PaCO_2_ exhibited an upward drift during the rewarming period, correlating with the temperature gradient rather than patient weight. Based on several correlations identified, we derived a formula to predict FiO_2_ based on patient weight, which would achieve the target PaO_2_ at the 1st blood gas series when using the FX05 oxygenator. *Conclusion*: We identified when and how the errors in calculating in-line PaO_2_ and PaCO_2_ occurred and developed several recommendations to minimize significant deviations from actual PaO_2_ and PaCO_2_ during CPB. Our results suggest that achieving acceptable PaO_2_ and PaCO_2_ calculations throughout CPB using a single universal formula for each, embedded in the Quantum System, is challenging due to the variety of oxygenators available, different patient sizes, and changing conditions during CPB.

## Introduction

During cardiopulmonary bypass (CPB), accurate and precise blood gas and electrolyte values are crucial for better patient care and outcomes. Laboratory blood analyzers, which employ an electrochemical method and are considered the gold standard, can provide blood gas analysis by sampling the blood intermittently and on demand. In contrast, continuous in-line blood gas monitoring (CILBGM) devices, which employ optical fluorescence and spectrophotometric methods and are considered trending devices, can continuously measure and display results [1–4].

CILBGM can provide real-time monitoring of patient acid-base and oxygenation status, which has been shown to be a valuable tool for more accurate blood gas management and improved patient outcomes [2, 3, 5–8]. The usage of CILBGM devices during CPB has increased over the years and is recommended as a standard of care [1, 9–11]. Since CILBGM devices employ a different method from laboratory blood analyzers, their accuracy and precision must meet acceptable targets when compared to laboratory blood analyzers. Clinical Laboratory Improvement Amendments (CLIA) guidelines provided new accuracy standards in 2025 for clinical laboratory testing. According to the CLIA guidelines, PO_2_ should be within ±15 mmHg or ±15% (greater), and PCO_2_ should be within ±5 mmHg or ±8% (greater) [12, 13].

CILGBM devices, such as the CDI system 500/550 (Terumo Medical Corporation, Somerset, NJ), measure PaO_2_ and PaCO_2,_ and B-Capta (Liva Nova, London, UK) measures PaO_2_ through direct contact with arterial blood using the optical fluorescence method. Several studies showed that PaO_2_ and PaCO_2_ of the CDI system 500 were acceptable (within the CLIA guideline) at the 1st blood gas series after the initiation of CPB with in vitro calibration using its own calibration gas and became more accurate and precise at the 2nd blood gas series with the first in vivo calibration [2–4, 8]. Without in vitro calibration, PaO_2_ and PaCO_2_ of the CDI system 500 at the 1st blood gas series were unacceptable. However, after the first in vivo calibration, they became acceptable at the 2nd blood gas series [14]. A study compared CDI system 500, B-Capta, and System M4 (Spectrum Medical, Gloucester, United Kingdom) for PaO_2_ and other parameters. Unlike the CDI system 500 and B-Capta, System M4 does not make direct contact with arterial blood and thus cannot measure but calculate PaO_2_. It showed that while CDI system 500 and B-Capta provided acceptable PaO_2_ at the 1st and 2nd blood gas series, System M4 exceeded the acceptable targets at both series [4].

Similar to System M4, the Quantum perfusion system with Quantum Ventilation_2_ (“Quantum System” hereafter; Spectrum Medical, Gloucester, United Kingdom) provides continuous in-line PaO_2_ and PaCO_2_ without direct contact with arterial blood. Thus, the Quantum System does not measure PaO_2_ or PaCO_2_. To circumvent this, Quantum System developed formulas to calculate PaO_2_ and PaCO_2_ with its own non-invasive sensors and embedded software. A small portion of expired gas of the oxygenator is constantly drawn from the active waste gas scavenging system to the sensor in Quantum Ventilation_2_ to measure the fraction of CO_2_ in the expired gas (FeCO_2_). In-line PaCO_2_ is calculated with their proprietary formula, which is heavily dependent on FeCO_2_. However, the calculated in-line PaO_2_ of Quantum System is complex and is mainly dependent on FiO_2_ and modified by other non-invasive measurements such as flow, SaO_2_, SvO_2_, FeCO_2_, hematocrit, temperature, and other variables. Several coefficient constants in the formula were determined by the best fitting of experimental data with at least two adult-size oxygenators (Personal Communication).

We have been using Quantum System with the FX05 oxygenator (Terumo Medical Corporation, Somerset, NJ) since August 2019 and observed that the calculated in-line PaO_2_ at the 1st blood gas series after the initiation of CPB has been significantly higher than PaO_2_ from the point-of-care (POC) blood analyzer (i-STAT, Abbott, Abbott Park, IL). We also noted that the calculated in-line PaO_2_ seems to drift upward during the rewarming and rewarmed periods. Similarly, the calculated in-line PaCO_2_ seems to drift upward during the rewarming period as well. These deviations prompted us to undertake a quality improvement initiative, focusing on identifying the degree of errors in both calculated in-line PaO_2_ and PaCO_2_ during the cooling and cooled periods, as well as during the rewarming and rewarmed periods. Since these are not the measured values, we expected a certain degree of errors, which should still be acceptable for safe practice. We were also interested in investigating whether any possible patterns or correlations exist between the errors and other factors, such as patient weight and temperature.

## Methods

### Patient population

We reviewed the EPIC (EPIC Systems, Verona, WI) electronic records of anesthesia and perfusion for 133 consecutive patients who underwent cardiac surgery with CPB using the Quantum System with the FX05 oxygenator at the Children’s Hospital of Philadelphia (CHOP) from January 3, 2023, to June 6, 2023. Quantum System, equipped with non-invasive sensors and embedded software, served as the CILBGM device. Patients were excluded based on criteria such as missing data, which is necessary to perform the analysis, heart or lung transplants, LVAD insertion, absence or interruption of cooling or rewarming, or if deceased. Fifty-two patients were excluded, resulting in 81 patients to be analyzed. Out of 81 patients, 23 were weight ≤ 4 kg, 43 were 4 kg < weight ≤ 8 kg, and 15 were 8 kg < weight ≤ 14 kg. Twenty-five patients had nadir nasopharyngeal temperature (Nadir T) during CPB ≥ 32 °C, 35 were 32 °C > Nadir T ≥ 24 °C, and 21 were Nadir T < 24 °C during CPB. Average CPB time was 136.8 ± 57.3 min and average cross-clamp time was 86.9 ± 47.2 min.

### Blood gas analysis and data collection

During CPB, pH-stat was used for blood gas management during the cooling and cooled periods and alpha-stat during the rewarming and rewarmed periods [15–17]. During the cooling and cooled periods, pH-stat results of blood gas analysis are recorded in our EPIC electronic record. During the rewarming and rewarmed periods, alpha-stat results are recorded. The cooling period indicates that the patient’s temperature is actively decreasing toward the target temperature, while the cooled period indicates that the patient’s temperature remained at the target temperature. The rewarming period indicates that patient temperature is actively increasing to reach the venous blood temperature of 36.5 °C, and the rewarmed period indicates that patient temperature is maintained at the venous blood temperature of 36.5 °C.

The sweep rate was between 0.5 and 1.5 LPM, which is relatively proportional to the patient’s weight, with an infusion of CO_2_ at a rate of 30–50 mL/min. CO_2_ field flooding was a surgeon’s preference. One of five surgeons routinely employed CO_2_ field flooding.

After the initiation of CPB and cooling, the 1st blood gas series was performed after 2–5 min of steady state CPB by drawing arterial and venous blood samples from the CPB circuit while pressing the “Capture All” key on the Quantum monitor at the same time. Blood gas analysis was performed with CD8 cartridges using iSTAT. After the blood gas analysis was completed, the “Sync” key was pressed to recall the stored values. The first in vivo calibration was performed by replacing the stored values with the iSTAT results. After the first in vivo calibration, arterial blood samples were drawn every 30 min or when necessary for the blood gas analysis, and in vivo calibrations were performed with every blood gas analysis.

During the cooling and cooled period, data from the first three blood gas series, if available, were collected. Many cases had short cooling and cooled periods or proceeded to deep hypothermic circulatory arrest (DHCA). Data is not collected anymore when DHCA is initiated. Thus, we were able to collect the 1st blood gas series from 81 patients, the 2nd blood gas series from 68, and 3rd blood gas series from 42 during the cooling or cooled period (Table 1).

**Table 1:** Average error in mmHg and %Error of the calculated in-line PaO_2_.

	Avg error (mmHg)	SD (mmHg)	Avg %error (%)	SD (%)	Patients (*n*)
1st gas series	117.0	64.5	48.3	41.3	81
2nd gas series	−35.8	67.4	−7.7	27.8	68
3rd gas series	6.5	21.6	3.4	10.0	42
4th gas series	41.0	56.9	24.7	37.0	78
5th gas series	45.1	56.0	26.6	38.3	51
4th+5th gas series	82.3	77.6	50.5	59.5	51

Avg: average; SD: standard deviation.

After the initiation of rewarming, the 4th blood gas series from 78 patients and the 5th blood gas series from 51 were collected (Table 1). Data from the Quantum System and anesthesia records corresponding to each blood gas series were manually extracted.

### Error in mmHg and %Error

Errors in mmHg and %Error for PaO_2_ and PaCO_2_ during the cooling and cooled periods (the 1st to 3rd blood gas series) were calculated using pH-stat values, while those during the rewarming and rewarmed periods (the 4th and 5th blood gas series) were calculated using alpha-stat values. The formulas are:

$$\begin{array}{c} \mathrm{Error}\;\mathrm{in}\;\mathrm{mmHg}=\mathrm{Quantum}\;\mathrm{System}\;\mathrm{value}-\mathrm{iSTAT}\;\mathrm{value}\;(\mathrm{mmHg})\\ \mathrm{\%Error}=\left[\left(\mathrm{Quantum}\;\mathrm{System}\;\mathrm{value}-\mathrm{iSTAT}\;\mathrm{value}\right)/\mathrm{iSTAT}\;\mathrm{value}\right]\times 100\left(\%\right). \end{array}$$

### Data analysis

Microsoft Excel Office 365 was employed for data input, calculations, scatter plots, fitted linear regression analysis, Bland-Altman analysis, and other statistical analyses, including correlation coefficient (*R*), bias, and limits of agreement (LOA) [18–20].

## Result

### Calculated in-line PaO_2_ is significantly overestimated before the first in vivo calibration

A significant error in the calculated in-line PaO_2_ of the Quantum System with the FX05 oxygenator was observed at the 1st blood gas series on CPB. The calculated in-line PaO_2_ is almost always overestimated compared to the measured PaO_2_ on iSTAT with an average %Error of 48.3 ± 41.3% (Table 1), which exceeds the acceptable target of  ±15% [12, 13]. These large errors and standard deviation (SD) indicate that the accuracy and precision of the calculated in-line PaO_2_ are unacceptable before the first in vivo calibration.

After the first in vivo calibration, at the 2nd blood gas series, the average error improved significantly to −7.7%, though SD remained high at ±27.8%. By the third blood gas series, the accuracy and precision further improved, with an average error of 3.4 ± 10% after the second in vivo calibration (Table 1).

As shown in the %Error, the calculated in-line PaO_2_ is significantly overestimated at the 1st blood gas series. The average error in mmHg was 117 mmHg, and the standard deviation was ±64.5 mmHg (Table 1). Over-estimation occurred in 99% of the patients, and 53% had over-estimation higher than 100 mmHg (Table 2). After the first and second in vivo calibrations, the over-estimation of the calculated in-line PaO_2_ was corrected as 4.4% and 0% of the patients had over-estimation higher than 100 mmHg at the 2nd and 3rd blood gas series, respectively (Table 2).

**Table 2:** Error distribution of the calculated in-line PaO_2_ and the correlation coefficient (*R*) between the error in mmHg and patient weight.

Error distribution (mmHg)	1st gas (% Pt)	2nd gas (% Pt)	3rd gas (% Pt)	4th gas (% Pt)	5th gas (% Pt)	4th + 5th (% Pt)
Error ≤ 0	1.2	82.4	45.2	21.8	13.7	15.7
0 < Error ≤ 100	45.7	13.2	54.8	62.8	72.5	41.2
100 < Error ≤ 200	39.5	4.4	0	15.4	13.7	37.3
200 < Error ≤ 300	12.3	0	0	0	0	3.9
300 < Error ≤ 400	1.2	0	0	0	0	2.0
*R* (error vs. weight)^$^	0.62	0.07	0.19	0.25	0.37	0.46

% Pt: percentage of patients, see Table 1 to find total patient numbers for each blood gas series; $: *R* value of the linear correlation analysis between the error in mmHg and patient weight.

### The error of the calculated in-line PaO_2_ at the 1st blood gas series strongly correlates with patient weight

**Figure 1:**
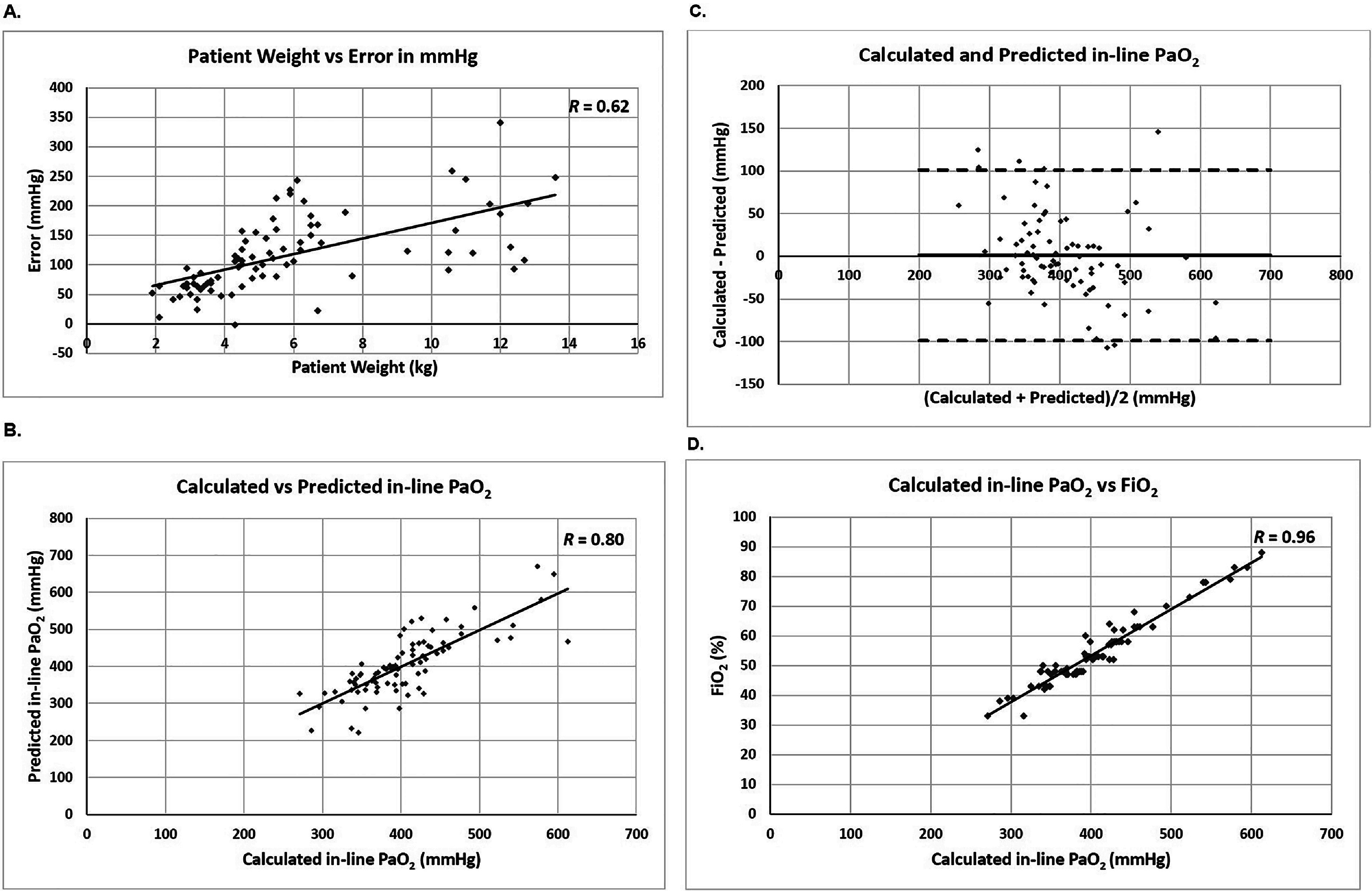
The error in the calculated in-line PaO_2_ of Quantum System at the 1st blood gas series has a strong correlation with the patient weight, which allows to derive a formula to compute the predicted in-line PaO_2_. (A) A scattered XY plot was drawn for patient weight (X-axis) and error in mmHg of the 1st blood gas series (Y-axis). The data was fitted into a linear regression line, showing a strong correlation. *R* value is shown in the upper right corner. (B) A scattered XY plot was drawn for the calculated in-line PaO_2_ of Quantum System (X-axis) and predicted in-line PaO_2_ computed with our formula (Y-axis). The data was fitted into a linear regression line, showing a very strong correlation. *R* value is shown in the upper right corner. (C) Bland-Altman analysis of the calculated and predicted in-line PaO_2_ shows strong agreement with a bias of 1 mmHg (bold solid line) and LOA of 101 and −99 mmHg (bold dashed lines). The X-axis represents the average of the calculated and predicted in-line PaO_2_, while the Y-axis represents the difference between the calculated and predicted in-line PaO_2_. (D) A scattered XY plot was drawn for the calculated in-line PaO_2_ (X-axis) and FiO_2_ (Y-axis) of Quantum System. The data was fitted into a linear regression line, showing a very strong correlation. *R* value is shown in the upper right corner.

As patient weight increases, oxygen consumption likely increases, which may contribute to the error if the calculated in-line PaO_2_ does not reflect the oxygen consumption correctly. Therefore, we examined whether the error in mmHg has a correlation with patient weight. As shown in Figure 1A, we found a strong linear correlation (*R* = 0.62, slope = 13, *Y* intercept = 40) between the error in mmHg at the 1st blood gas series and patient weight. The error in mmHg increases as patient weight increases. However, after the first in vivo calibration, this correlation disappeared at the 2nd blood gas series (*R* = 0.07) and at the 3rd blood gas series (*R* = 0.19) after the second in vivo calibration (Table 2).

From the strong linear correlation at the 1st blood gas series, we derived a formula to compute a predicted in-line PaO_2_ based on the measured PaO_2_ at the 1st blood gas series:

$$\mathrm{Predicted}\;\mathrm{in}‐\mathrm{line}\;{\mathrm{PaO}}_{2}\;\left(\mathrm{mmHg}\right)=\mathrm{measured}\;{\mathrm{PaO}}_{2}+13\times \mathrm{Weight}+40.$$

Using this formula, we determined the predicted in-line PaO_2_ of all 81 patients with the measured PaO_2_ and patient weight and compared it to the calculated in-line PaO_2_ of the Quantum System. We found a very strong linear correlation between the calculated and predicted in-line PaO_2_ (*R* = 0.8, slope = 1.0, *Y* intercept = 2.3, see Figure 1B). The Bland-Altman analysis shows that the bias (average difference) is 1 mmHg and LOA (1.96 × SD, 95% of data is within the LOA) is 101 and −99 mmHg between the calculated and predicted in-line PaO_2_ (Figure 1C) [19, 21–23].

Based on the very strong linear correlation between the calculated and predicted in-line PaO2, we can use our formula to calculate the predicted in-line PaO_2_ to achieve a target PaO_2_ at the 1st blood gas series on CPB. This can be done by substituting the measured PaO_2_ with a target PaO_2_ in the formula:

$$\mathrm{Predicted}\;\mathrm{in}‐\mathrm{line}\;{\mathrm{PaO}}_{2}=\mathrm{target}\;{\mathrm{PaO}}_{2}+13\times \mathrm{Weight}+40.$$

For example, if a target PaO_2_ is 250 mmHg with a 10 kg patient, the predicted in-line PaO_2_ is 420 mmHg. This suggests that if we set FiO_2_ to achieve the calculated in-line PaO_2_ of 420 mmHg when we perform the 1st blood gas series on CPB, the actual PaO_2_ is approximately 250 mmHg.

### Calculated in-line PaO_2_ is essentially determined by FiO_2_ before the first in vivo calibration

The error observed at the 1st blood gas series increased as patient weight increased, suggesting that the impact of oxygen consumption on the in-line PaO_2_ calculation is minimal prior to the first in vivo calibration. Therefore, we investigated how strongly the calculated in-line PaO_2_ is dependent on FiO_2_ before the first in vivo calibration. Almost a perfect linear correlation exists between the calculated in-line PaO_2_ and FiO_2_ at the 1st blood gas series (*R* = 0.96; Figure 1D). However, this correlation diminished significantly following the first in vivo calibration, showing low correlation values in the subsequent blood gas series, specifically *R* = 0.11 at the 2nd series and *R* = 0.18 at the 3rd series (Data not shown).

Utilizing this very strong linear correlation, we derived a formula to predict FiO_2_ to achieve a predicted in-line PaO_2_ at the 1st blood gas series:

$$\mathrm{Predicted}\;{\mathrm{FiO}}_{2}=0.16\;\times \;\mathrm{predicted}\;\mathrm{in}‐\mathrm{line}\;{\mathrm{PaO}}_{2}-9.$$

Then, by using our predicted in-line PaO_2_ formula, we can derive another formula to predict FiO_2_ to achieve a target PaO_2_ at the 1st blood gas series:

$$\begin{array}{c} \mathrm{Predicted}\;{\mathrm{FiO}}_{2}=0.16\times \left(\mathrm{target}\;{\mathrm{PaO}}_{2}+13\times \mathrm{Weight}+40\right)-9\\ =\;0.16\times \mathrm{target}\;{\mathrm{PaO}}_{2}+2.1\times \mathrm{Weight}-3. \end{array}$$

For example, to achieve a target PaO_2_ of 250 mmHg with a 10 kg patient at the 1st blood gas series, the predicted FiO_2_ is 58%, which is expected to result in the calculated in-line PaO_2_ of approximately 420 mmHg and the measured PaO_2_ of around 250 mmHg.

### Calculated in-line PaO_2_ drifts upward significantly during the rewarming and rewarmed periods

We showed that after in vivo calibrations, the calculated in-line PaO_2_ became acceptable during the cooling and cooled periods. However, the errors were significantly overestimated again during the rewarming and rewarmed periods. At least two blood gases (the 4th and 5th blood gas series) of 51 patients were measured during the rewarming and rewarmed periods. The errors in mmHg at the 4th and 5th blood gases were calculated separately and combined. The combined errors of calculated in-line PaO_2_ were over-estimated in 84% of the patients (Table 2) with the average %Error of 50.5 ± 59.5% (Table 1), which is beyond the acceptable target. These large %Error and SD indicate that the calculated in-line PaO_2_ drifted upward significantly during the rewarming and rewarmed periods.

**Figure 2:**
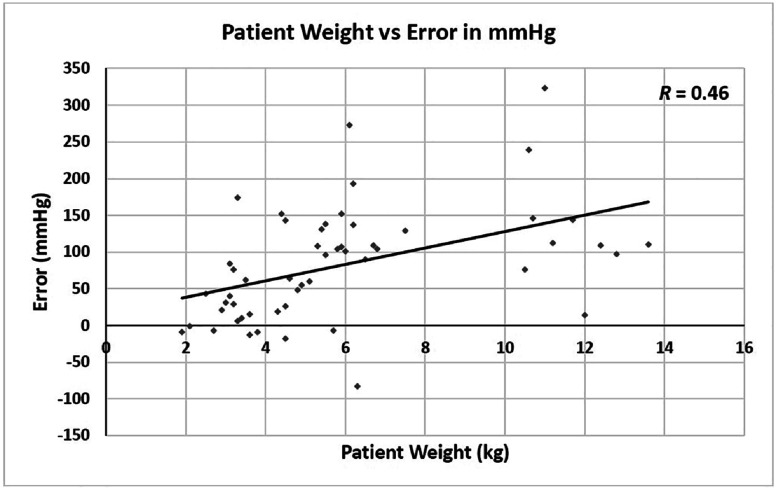
The calculated in-line PaO_2_ of Quantum System drifts upward during the rewarming and rewarmed periods, showing a moderate correlation to patient weight. A scattered XY plot was drawn for patient weight (X-axis) and combined error in mmHg of the 4th and 5th blood gas series (Y-axis). The data was fitted into a linear regression line, showing a moderate correlation. *R* value is shown in the upper right corner.

The average combined error in mmHg was 82.3 mmHg and SD was ±77.6 mmHg (Table 1). Over-estimation higher than 100 mmHg occurred in 43% of the patients (Table 2). As shown in Figure 2, there is a moderate linear correlation (*R* = 0.46) between the combined error in mmHg at the 4th and 5th blood gas series and patient weight.

### Calculated in-line PaCO_2_ is acceptable without in vivo calibration during the cooling and cooled periods

We found that the error of calculated in-line PaCO_2_ is acceptable at the 1st blood gas series without in vivo calibration and at the 2nd and 3rd blood gas series during the cooling and cooled periods (±5 mmHg or ±8% greater; Table 3). This is likely due to the fact that the in-line PaCO_2_ calculation is largely dependent on the actual measurement of FeCO_2_ of the oxygenator. Nevertheless, SD is largest at the 1st blood gas series and becomes smaller at the 2nd and 3rd blood gas series with each in vivo calibration (Table 3). Errors between 0 and 5 mmHg were observed in 73.2% of the patients at the 3rd blood gas series, compared to 18.5% and 42.6% at the 1st and 2nd blood gas series, respectively (Table 4).

**Table 3:** Average error in mmHg and %Error of the calculated in-line PaCO_2_

	Avg error (mmHg)	SD (mmHg)	Avg %Error (%)	SD (%)	Patients (*n*)
1st gas series	−3.6	6.4	−7.8	14.2	81
2nd gas series	0.2	4.8	0.9	11.5	68
3rd gas series	1.1	1.8	2.8	4.5	42
4th gas series	4.4	5.4	11.5	15.7	77
5th gas series	−0.7	3.5	−1.4	8.1	50
4th+5th gas series	3.9	6.8	10.5	18.6	50

Avg: average; SD: standard deviation.

**Table 4:** Error distribution of the calculated in-line PaCO_2_ and the correlation coefficient (*R*) between the error in mmHg and patient weight.

Error distributions (mmHg)	1st gas (%)	2nd gas (%)	3rd gas (%)	4th gas (%)	5th gas (%)	4th + 5th (%)
Error ≤ 0	75.3	44.1	26.8	18.2	66.0	28.0
0 < Error ≤ 5	18.5	42.6	73.2	46.8	28.0	40.0
5 < Error ≤ 10	2.5	13.2	0	23.4	6.0	16.0
10 < Error ≤ 15	2.5	0	0	5.2	0	6.0
15 < Error ≤ 20	1.2	0	0	5.2	0	8.0
20 < Error ≤ 25	0	0	0	1.3	0	2.0
*R* (error vs. weight)^$^	0.22	0.17	−0.10	−0.24	−0.14	−0.21

%: percentage of patients, see Table 3 to find total patient numbers for each blood gas series; $: *R* value of the linear correlation analysis between the error in mmHg and patient weight.

### Calculated in-line PaCO_2_ drifted upward during the rewarming period, correlating with the temperature gradient

While the calculated in-line PaCO_2_ remained acceptable during the cooling and cooled periods, it drifted upward during the rewarming period. At the 4th blood gas series, which is the first blood gas measured after the rewarming was initiated, the error of the calculated in-line PaCO_2_ was 4.4 ± 5.4 mmHg or 11.5 ± 15.7% (Table 3). At the 4th blood gas series, 35% of the patients had errors higher than 5 mmHg compared to 0% at the 3rd blood gas series (Table 4). The error became acceptable at the 5th blood gas series following the in vivo calibration after the 4th blood gas series (Table 3).

**Figure 3:**
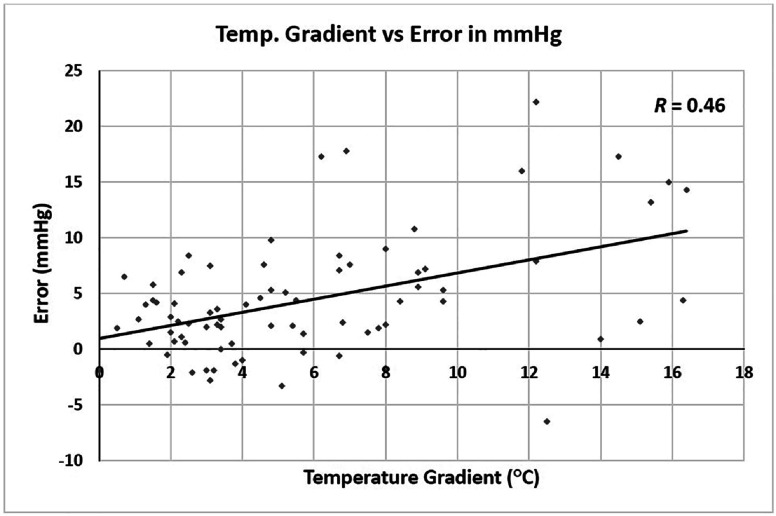
The calculated in-line PaCO_2_ of Quantum System drifts upward during the rewarming period, correlating with the temperature gradient. The temperature gradient is defined as the patient’s nasopharyngeal temperature at the 4th blood gas series minus nadir T during CPB. A scattered XY plot was drawn for the temperature gradient (X-axis) and error in mmHg of the 4th blood gas series (Y-axis). The data was fitted into a linear regression line, demonstrating a moderate correlation. *R* value is shown in the upper right corner.

Notably, the errors of calculated in-line PaCO_2_ showed very weak or weak correlations with patient weight across all blood gas series (see *R* values in Table 4). However, a moderate correlation was identified between the error in mmHg and temperature gradient, defined as the difference between the patient’s nasopharyngeal temperature at the 4th blood gas series and nadir T during CPB, with an *R* value of 0.46 (Figure 3).

## Discussion

In this retrospective study, we demonstrated that the calculated in-line PaO_2_ of the Quantum System with the FX05 oxygenator is unacceptable at the 1st blood gas series. The average error was 117 mmHg, and the average %Error was 48.3%, which exceeds the acceptable target (Table 1). Notably, the calculated in-line PaO_2_ at the 1st blood gas series is almost perfectly correlated with FiO_2_ (Figure 1D), indicating other factors such as the flow, SaO_2_, SvO_2_, FeCO_2_, temperature, and other variables have minimal impact on PaO_2_ calculation prior to the first in vivo calibration.

Following the first in vivo calibration, the calculated in-line PaO_2_ at the 2nd blood gas series became acceptable, although it exhibited a high SD (Table 1). After the second in vivo calibration, the error and SD were reduced, demonstrating improved accuracy and precision with each in vivo calibration. This suggests that it is a better practice to use “Capture All/Sync” each time with the blood gas analysis when using the Quantum System.

However, when rewarming began, the calculated in-line PaO_2_ drifted upward, leading to unacceptable error in mmHg and %Error at two consecutive blood gas series (4th and 5th in Table 1). In 51 patients, when two consecutive blood gas series were performed during the rewarming and rewarmed periods, the combined average error in mmHg was 82.3 mmHg and the average %Error was 50.5% (Table 1). This suggests that the in-line PaO_2_ calculation may underestimate oxygen consumption during the rewarming and rewarmed periods.

Quantum System offers an autoregulation function of PaO_2_ and PaCO_2_, which relies heavily on the accuracy of the calculated in-line PaO_2_ and PaCO_2_. Our findings indicate that the autoregulation function of PaO_2_ should not be used before the first in vivo calibration and during the rewarming and rewarmed periods, at least when using the FX05 oxygenator. Since the calculated in-line PaO_2_ is significantly higher than the actual PaO_2_ during these periods, it is possible that PaO_2_ can become dangerously low with the autoregulation function.

We identified a significant error in the calculated in-line PaO_2_ exists at the 1st blood gas series, strongly correlated with patient weight (Table 2 and Figure 1A). The combined errors during the rewarming and rewarmed periods moderately correlated with patient weight (*R* = 0.46, Table 2, Figure 2). A very strong correlation between the calculated in-line PaO_2_ and FiO_2_ at the 1st blood gas series was observed (Figure 1D). Using these strong correlations, we derived a formula to predict FiO_2_ based on patient weight to achieve a target PaO_2_ at the 1st blood gas series (see Results). We applied this formula in 15 cases, aiming for a target PaO_2_ of 250 mmHg for validation of the formula. The measured PaO_2_ with iSTAT ranged from 180 to 290 mmHg, with an average and standard deviation of 243 ± 39 mmHg. In contrast, the calculated in-line PaO_2_ of Quantum System ranged from 329 to 394 mmHg, with an average and standard deviation of 355 ± 20 mmHg (data not shown). The measured PaO_2_ were all within LOA, suggesting the formula effectively predicts FiO_2_ for the target PaO_2_ at the 1st blood gas series. Table 5 provides the predicted FiO_2_ based on patient weight for achieving the target PaO_2_ of 250 mmHg and the predicted in-line PaO_2_ corresponding to the predicted FiO_2_ at the 1st blood gas series using the FX05 oxygenator.

**Table 5:** Predicted FiO_2_ to achieve the target PaO_2_ of 250 mmHg and the predicted in-line PaO_2_ corresponding to the predicted FiO_2_.

Weight (kg)	Target PaO_2_ (mmHg)	Predicted FiO_2_ (%)	Predicted in-line PaO_2_ (mmHg)
2	250	41	316
3	250	43	329
4	250	45	342
5	250	48	355
6	250	50	368
7	250	52	381
8	250	54	394
9	250	56	407
10	250	58	420
11	250	60	433
12	250	62	446
13	250	64	459
14	250	66	472

Given the potential for dangerously low actual PaO_2_ during the rewarming and rewarmed periods despite the high calculated in-line PaO_2_ of the Quantum System, we emphasize monitoring the in-line SaO_2_ during these periods. The in-line SaO_2_ of the Quantum System, measured using the spectrophotometric method, has proven accurate throughout CPB [24]. We recommend increasing FiO_2_ if SaO_2_ decreases by more than 3–4% after the initiation of rewarming and conducting a blood gas analysis to calibrate the calculated in-line PaO_2_.

The calculated in-line PaCO_2_ of the Quantum System is acceptable during the cooling and cooled periods without the first in vivo calibration, although SD is largest at the 1st blood gas series and decreases with each calibration (Table 3). The proportion of larger error distribution diminishes as well (Table 4). These observations indicate improved precision with each in vivo calibration, supporting the use of “Capture All/Sync” with each blood gas analysis.

During the rewarming period, the calculated in-line PaCO_2_ drifted upward (Table 4), unrelated to patient weight but correlated with the temperature gradient (Figure 3). This likely occurs as colder venous blood encounters the warmer oxygenator, leading to transiently more CO_2_ expiration at the oxygenator, causing upward drift, as the calculated in-line PaCO_2_ relies on FeCO_2_ measurement.

We initiated this quality improvement initiative with the question, “Is continuous in-line blood gas monitoring reliable during cardiopulmonary bypass when PaO_2_ and PaCO_2_ are calculated rather than measured?” Since Quantum System does not measure PaO_2_ or PaCO_2_, it may not need to comply with CLIA guidelines. However, as a claimed CILBGM device, it should provide values within acceptable targets throughout CPB. We demonstrated that the calculated in-line PaO_2_ of the Quantum System is unreliable before the first in vivo calibration and during the rewarming and rewarmed periods. This is likely due to reliance on a single universal formula for different oxygenators, patient sizes, conditions, and surgical procedures without measuring actual PaO_2_. By analyzing our data of the Quantum System with the FX05 oxygenator, we identified when and how errors in the calculated in-line PaO_2_ and PaCO_2_ occur, leading to recommendations for minimizing significant deviations from actual values. However, this does not align with expectations for a CILBGM device. Specific formulas may need development for each oxygenator and varying conditions during CPB, such as different patient sizes, before the first in vivo calibration, cooling, rewarming, etc. The calculated in-line PaO_2_ and PaCO_2_ should meet the acceptable targets to be reliable throughout CPB as a CILBGM device.

### Limitations

The findings in this study are limited to the FX05 oxygenator, and the formulas and recommendations provided are specific to this model. We observed similar patterns of error with the FX15 oxygenator, indicating the need for further investigation. Additionally, adult-size oxygenators may warrant similar investigation to understand and mitigate potential errors.

iSTAT is a POC blood analyzer, which has demonstrated varying levels of deviation in PO_2_ measurement compared to benchtop or laboratory gas analyzers, depending on the study and patient populations. While most studies indicate good correlations or minimal deviations [25-28], there is a notable exception in a study involving lung donors, which revealed relatively large deviations [29].

## Data Availability

The data supporting the findings of this study are not publicly available. It may be available upon request to the corresponding author.

## References

[1] Stammers AH. Monitoring controversies during cardiopulmonary bypass: how far have we come? Perfusion. 1998;13:35–43. 9500247 10.1177/026765919801300105

[2] Southworth R, Sutton R, Mize S, et al. Clinical evaluation of a new in-line continuous blood gas monitor. J Extra Corpor Technol. 1998;30:166–170. 10537576

[3] Trowbridge CC, Vasquez M, Stammers AH, et al. The effects of continuous blood gas monitoring during cardiopulmonary bypass: a prospective, randomized study – part I. J Extra Corpor Technol. 2000;32:120–128. 11146955

[4] van Hoeven M, Overdevest E, Curvers J, van Heugten H. A comparison of continuous blood gas monitors during cardiopulmonary bypass LivaNova B-Capta, Terumo CDI 500, Spectrum Medical M4. Perfusion. 2023;38:740–746. 35285344 10.1177/02676591221080524

[5] Swan H, Sanchez M, Tyndall CM, Koch C. Quality control of perfusion: monitoring venous blood oxygen tension to prevent hypoxic acidosis. J Thorac Cardiovasc Surg. 1990;99:868–872. 2329825

[6] Trowbridge CC, Vasquez M, Stammers AH, et al. The effects of continuous blood gas monitoring during cardiopulmonary bypass: a prospective, randomized study – part II. J Extra Corpor Technol. 2000;32:129–137. 11146956

[7] Fried DW, Leo JJ, Mattioni GJ, et al. CDI blood parameter monitoring system 500 – a new tool for the clinical perfusionist. J Extra Corpor Technol. 2000;32:25–30. 10947620

[8] Ottens J, Tuble SC, Sanderson AJ, Knight JL, Baker RA. Improving cardiopulmonary bypass: does continuous blood gas monitoring have a role to play? J Extra Corpor Technol. 2010;42:191–198. 21114221 PMC4679958

[9] Stammers AH, Mejak BL, Rauch ED, Vang SN, Viessman TW. Factors affecting perfusionists’ decisions on equipment utilization: results of a United States survey. J Extra Corpor Technol. 2000;32:4–10. 10947623

[10] Baker RA, Willcox TW. Australian and New Zealand perfusion survey: equipment and monitoring. J Extra Corpor Technol. 2006;38:220–229. 17089508 PMC4680813

[11] Walcƶak A, Klein T, Voss J, et al. International pediatric perfusion practice: 2016 survey results. J Extra Corpor Technol. 2021;53:7–26. 33814602 10.1182/ject-2000033 PMC7995632

[12] Rivers PA, Dobalian A, Germinario FA. A review and analysis of the clinical laboratory improvement amendment of 1988: compliance plans and enforcement policy. Health Care Manage R. 2005;30:93–102. 10.1097/00004010-200504000-00003 15923911

[13] Bennett SA, Conn CM, Gill HE, et al. Regulatory requirements for laboratory developed tests in the United States. J Immunol Methods. 2025;537:113813. 39880119 10.1016/j.jim.2025.113813

[14] Bellaiche AL, Nielsen PF, Brantlov S, Møller MB, Winterdahl M. Clinical evaluation of the accuracy and precision of the CDI 500 in-line blood gas monitor with and without gas calibration. J Extra Corpor Technol. 2011;43:53–57. 21848172 PMC4680023

[15] Jonas RA, Bellinger DC, Rappaport LA, Wernovsky G, Hickey PR, Farrell DM, Newburger JW. Relation of pH strategy and developmental outcome after hypothermic circulatory arrest. J Thorac Cardiovasc Surg. 1993;106:362–368. 8341077

[16] Hiramatsu T, Miura T, Forbess JM, et al. pH strategies and cerebral energetics before and after circulatory arrest. J Thorac Cardiovasc Surg. 1995;109:948–58. 7739257 10.1016/S0022-5223(95)70321-7

[17] du Plessis AJ, Jonas RA, Wypij D, et al. Perioperative effects of alpha-stat versus pH-stat strategies for deep hypothermic cardiopulmonary bypass in infants. J Thorac Cardiovasc Surg. 1997;114:991–1001. 9434694 10.1016/S0022-5223(97)70013-8

[18] Lee MH, Gisnarian CJ, Shann KG. Improved estimation of total blood volume can provide a reliable prediction of dilutional hematocrit and oxygen delivery during cardiopulmonary bypass. J Extra Corpor Technol. 2019;5:67–72. PMC6586263 31239578

[19] Lee MH, Riley W. Shann KG Can the minimum protamine dose to neutralize heparin at the completion of cardiopulmonary bypass be significantly lower than the conventional practice? J Extra Corpor Technol. 2021;53:170–176. 34658407 10.1182/ject-2100023 PMC8499638

[20] Lee MH, Riley W. Factors associated with errors in the heparin dose response test: recommendations to improve individualized heparin management in cardiopulmonary bypass. Perfusion. 2021;36:513–523. 32909506 10.1177/0267659120952977

[21] Altman DG, Bland JM. Improving doctors’ understanding of statistics. J R Stat Soc Ser A Stat Soc. 1991;154:223–248.

[22] Giavarina D. Understanding Bland Altman analysis. Biochem Med. 2015;25:141–151. 10.11613/BM.2015.015 PMC4470095 26110027

[23] Bland JM, Altman DG. Measuring agreement in method comparison studies. Stat Methods Med Res. 1999;8:135–160. 10501650 10.1177/096228029900800204

[24] Reagor JA, Gao Z, Tweddell JS. Spectrum medical quantum or Terumo CDI 500: which device measures hemoglobin and oxygen saturation most accurately when compared to a benchtop blood analyzer? J Extra Corpor Technol. 2021;53:181–185. 34658409 10.1182/ject-2100003 PMC8499634

[25] Steinfelder-Visscher J, Teerenstra S, Gunnewiek JM, Weerwind PW. Evaluation of the i-STAT point-of-care analyzer in critically ill adult patients. J Extra Corpor Technol. 2008;40:57–60. 18389666 PMC4680657

[26] Indrasari ND, Wonohutomo JP, Sukartini N. Comparison of point‐of‐care and central laboratory analyzers for blood gas and lactate measurements. J Clin Lab Anal. 2019;33:e22885. 30924550 10.1002/jcla.22885 PMC6595289

[27] Kantekin ÇU, Ercan M, Oğuz EF, et al. Evaluation of the i-STAT Blood Gas Analysis System in Cardiovascular Surgery. Med LabTechnol. 2018;4:35–42.

[28] Jawa A, Motara F, Moolla M, Laher AE. A comparative assessment of the nova stat profile prime plus^®^ critical care analyzer. Cureus. 2020;21:12. 10.7759/cureus.9932 PMC7505621 32968593

[29] Marklin GF, Bresler R, Dhar R. Point-of-care blood gas analyzers have an impact on the acceptance of donor lungs for transplantation. Scand J Clin Lab Invest. 2020;80:623–629. 32955374 10.1080/00365513.2020.1821395

